# Dung beetle assemblage changes along a chronosequence in a recovering tropical dry forest

**DOI:** 10.1371/journal.pone.0337635

**Published:** 2025-12-04

**Authors:** Jibram León, Daniel González-Tokman, Teresa Castillo-Burguete, Víctor Manuel Vidal-Martínez, José Luis Hernández-Stefanoni, Carlos N. Ibarra-Cerdeña

**Affiliations:** 1 Departamento de Ecología Humana, Cinvestav, Antigua Carretera a Progreso, Mérida, Yucatán, México; 2 Instituto de Ecología A. C., Xalapa, Veracruz, México; 3 Departamento de Recursos del Mar, Cinvestav, Antigua Carretera a Progreso, Mérida, Yucatán, México; 4 Instituto Mexicano de Pesca y Acuacultura Sustentables, CDMX, México; 5 Centro de investigación Científica de Yucatán (CICY), Mérida, Yucatán, México; Instituto Federal de Educacao Ciencia e Tecnologia Goiano - Campus Urutai, BRAZIL

## Abstract

Tropical dry forests are among the most threatened ecosystems globally, facing extensive degradation from land-use change. Understanding how biodiversity responds during forest regeneration is critical for conservation and sustainable land management. We assessed the community composition and functional diversity of dung beetles (Scarabaeinae) across a forest recovery chronosequence (1–100 years) in the Yucatán Peninsula. The study area is characterized by traditional Mayan agroforestry systems that shape the landscape. 90 pitfall traps were set up across six forest age classes and were collected 6,605 individuals from 23 species and 13 genera. Dung beetle species richness, biomass, and abundance were significantly associated with forest structural, and diversity metrics. Forest Shannon entropy (*H*′), inverse Simpson concentration (*IF*₀, ₂), and aboveground biomass emerged as strong predictors of community attributes. Abundance and biomass responses varied by functional group: small diurnal rollers (SRD) increased with land-use intensity, while large nocturnal rollers (LRN), large diurnal tunnellers (LTD), and small nocturnal tunnellers (STN) declined sharply from mature forests to early successional stages and agricultural areas. Species richness (^0^*D*) peaked in early to intermediate successional stages (5–20 years), whereas dominant species diversity (^2^*D*) was highest in mixed-use forests under moderate disturbance. Distance-based redundancy analysis (db-RDA) and multi-model inference revealed that forest attributes—including DBH, aboveground biomass, canopy openness, and litter depth—jointly explained 48.7% of the variation in dung beetle assemblage structure (p < 0.001). Litter volume was positively correlated with species richness (adj. R^2^ = 0.76), and IF₀,₂ was a key predictor of biomass (adj. R^2^ = 0.62). Our findings reveal threshold-based and trait-mediated responses of dung beetle assemblages to forest succession, highlighting the ecological importance of bioculturally managed landscapes. These results underscore the role of secondary forests in maintaining biodiversity and ecosystem functions, supporting their conservation as vital components of tropical dry forest recovery.

## Introduction

Land-use change is a leading driver of biodiversity loss and ecosystem degradation, particularly in tropical dry forests, which are globally significant yet among the most threatened tropical forest ecosystems [1,2]. The conversion of continuous forests into traditional agricultural systems induces substantial alterations in habitat structure, resource availability, and microclimatic conditions, which in turn shape the composition and functional dynamics of biological communities [3]. Common patterns resulting from these changes include a marked decline in specialist and forest-dependent species, often replaced by generalist species that are more tolerant to open or disturbed environments [4,5]. Functional diversity frequently decreases as traits associated with forest specialization, such as large body size or specific nesting strategies, become less common, while traits favoring high dispersal capacity or resilience to disturbance become dominant [6]. These shifts can lead to biotic homogenization and a reduction in ecosystem functions, particularly in highly fragmented or intensively managed landscapes [7].

Chronosequences are specific applications of the “space-for-time substitution” framework, a widely used approach for assessing the ecological impacts of land-use change and succession dynamics [8,9]. This framework assumes that spatially distinct sites representing different stages of land-use change can serve as proxies for temporal processes, enabling researchers to infer patterns of biodiversity and ecosystem function without the need for long-term monitoring [10]. Chronosequences expand on the space-for-time substitution concept by systematically selecting sites that represent different stages of land-use history or ecological succession—from relatively undisturbed forests to highly modified areas—with the goal of reconstructing the temporal trajectories of forest use, recovery, and associated ecological processes [11]. This approach is particularly valuable in tropical dry forests, where logistical and financial constraints often preclude long-term ecological monitoring based on permanent plots or repeated measurements over time [12]. By using chronosequences, researchers can empirically test ecological hypotheses, such as how habitat filtering, biotic homogenization, or functional trait shifts unfold over time as a result of land-use change [13]. These designs are critical for disentangling the mechanisms driving community assembly and for identifying thresholds or tipping points in biodiversity and ecosystem functionality under anthropogenic pressures.

Traditionally, chronosequence studies assess ecological succession by comparing sites that span a gradient from undisturbed to highly disturbed conditions [14]. However, more recent research has emphasized the use of naturally recovered forests as reference sites to provide more ecologically realistic benchmarks of successional trajectories. For example, Letcher and Chazdon [15] examined biomass accumulation and plant species richness in a Costa Rican chronosequence of naturally recovered tropical forests. They found that secondary forests older than 30 years converged toward old-growth forest values in terms of structure and species composition. Similarly, Ferreira et al. [16], working in a Brazilian tropical dry forest, reported that tree species in early successional stages—also within naturally recovered forests—invested more heavily in conservative traits such as chemical defenses and desiccation tolerance compared to species in later stages. These studies underscore the value of incorporating naturally recovered forests into chronosequence designs to better capture the ecological processes that underpin forest recovery.

Dung beetles (Coleoptera: Scarabaeinae) are widely recognized as effective model organisms for studying species responses to land-use change due to their sensitivity to environmental conditions, and their critical ecological roles, such as dung burial, nutrient cycling, secondary seed dispersal, and soil aeration [17]. Numerous studies have employed dung beetles to investigate species-disturbance relationships [18], including those using chronosequences in tropical forests [19]. For example, Braga et al. [7] demonstrated that dung beetle assemblages in a chronosequence of secondary forests in the Brazilian Atlantic Forest progressively recovered species richness, and functional diversity with forest age, though the composition of forest specialists remained incomplete. A similar trend was also observed in the Amazon, where Noriega et al. [19] reported that species richness increased with time since forest recovery, but also emphasized that beta diversity was best explained by nestedness, driven by the appearance of species and functional groups in later successional stages, thereby supporting non-deterministic changes in dung beetle assemblage structure along forest succession. Similarly, Gardner et al. [5] found that in Amazonian landscapes, dung beetle diversity, and ecosystem functions, such as dung burial, were significantly reduced in agricultural matrices compared to continuous forest, but partially recovered in older secondary forests, supporting the importance of landscape connectivity, and habitat quality. Larsen et al. [20] highlighted the trait-dependent nature of community disassembly, showing that species with specific ecological traits, such as large body size, were more vulnerable to fragmentation. Complementing these findings, Noriega et al. [19] synthesized global patterns and identified declines in species richness and functional diversity as consistent outcomes of habitat degradation and fragmentation.

Despite these advances, critical questions about dung beetle assemblage structure and functional diversity remain unresolved. Key gaps include evaluations of dung beetle assemblages in dry forests, and understanding how specific functional traits, such as body size, diel activity, or nesting behavior, are affected by different types of disturbance [20,21]. The mechanisms driving the turnover of specialist versus generalist species along land-use gradients also require further exploration particularly in disentangling the relative contributions of environmental filtering and dispersal limitation [5,7], as predicted to occur between different ecoregions [22]. Furthermore, the role of historical land-use intensity and landscape configuration in shaping functional trait diversity and community assembly processes remains unclear [13,19]. Addressing these questions is crucial for advancing theoretical frameworks of biodiversity response to land-use change and for guiding effective conservation strategies in tropical ecosystems.

In the Yucatan Peninsula, the tropical dry forest has evolved through a process shaped by both natural dynamics and human interventions [21]. Historical patterns of forest change date back to the Classic Period of Mayan Occupation, during which high population densities fostered the development of sophisticated agroforestry systems [23]. These systems are thought to reflect sustainable management practices derived from empirical observations of plant phenology and an understanding of vegetation dynamics under varying climatic conditions [23,24]. One prominent example is the Mayan milpa system, a method of annual crop rotation that supports intermediate stages of forest succession and eventually facilitates the restoration of mature secondary forests with high species richness [25,26]. This approach relies on shifting agriculture with slash-and-burn practices to enhance nutrient recycling and accelerate ecological succession, reflecting a comprehensive understanding of soil dynamics [27]. However, the expansion of agriculture under intensified practices may interrupt successional trajectories and impair associated ecosystem services [28]. This underscores the need for studies that disentangle and predict the consequences of contemporary land-use changes on biodiversity maintenance. Such research is crucial for understanding the role of human intervention strategies in sustaining ecosystems whose traditional management has historically ensured their resilience and persistence [29–31].

Here in, we aimed to assess the changes in the assemblages of dung beetles along a chronosequence of Maya Forest to uncover the association between vegetation structure and diversity, microclimate and forest age on species and functional diversity. We expect dung beetle assemblages to respond to forest succession in patterns that reflect the degree of habitat modification. Specifically, we predict: (i) a linear negative correlation between dung beetle community attributes—such as species richness, biomass, and functional group abundance—and changes in forest community characteristics, including vegetation structure, environmental conditions, biodiversity, and productivity. This relationship is anticipated because increasing anthropogenic intensity—stemming from productive land-use activities such as beekeeping, selective logging, plantations, and cattle ranching—progressively reduces habitat heterogeneity. Such reductions are most pronounced in recently modified land uses compared to the more complex and structurally diverse habitats of mature, naturally recovered forests. As habitat heterogeneity declines, we expect a corresponding decrease in species richness and changes in the composition of dung beetle assemblages; (ii) a stepwise or threshold response function, wherein dung beetle assemblage attributes undergo abrupt shifts in response to specific changes in successional stages, indicating that anthropogenic disturbances create persistent ecological filters that alter habitat heterogeneity and disrupt community assembly processes; (iii) a spatially localized negative correlation—at the plot level—between habitat degradation and dung beetle assemblage attributes, coupled with a broader positive effect across successional stages. In this scenario, site-specific disturbances initially reduce species richness, biomass, and functional diversity. However, as forest recovery progresses at the landscape scale, functionally diverse dung beetle assemblages gradually recolonize, driven by improved habitat quality and connectivity. These expected patterns highlight the complex interplay between land-use change, habitat structure, and dung beetle community dynamics, providing insight into the mechanisms driving biodiversity shifts in recovering tropical dry forests.

## Materials and methods

### Permits and site access

Access to the Kaxil Kiuic Biocultural Reserve and permission to conduct sampling were granted by the reserve’s administration. Permission to sample in the agricultural sites was provided by the respective landowners.

### Study area

The study was conducted in the towns of Yaxhachén, X-Kobehaltún, and Santa Rita, located within the municipality of Oxkutzcab in the southern region of Yucatán, Mexico. Sampling sites were established in the Kaxil Kiuic Biocultural Reserve (KKBR) (20° 06’ 10.8"N; 89° 33’ 43.2"W) at 75–150 m a.s.l. The reserve covers 1,650 ha of low and medium subdeciduous forest with an average canopy height of 25–30 m. Additional sampling was conducted in agricultural buffer zones adjacent to the reserve. The region experiences an average annual temperature of 26–30 °C, with annual precipitation ranging from 1,000–1,100 mm. Relative humidity averages 65.99% ± 2.81 during the warm-dry season (March–July) and 80.19% ± 1.73 during the warm-humid season (August–October) [32].

The KKBR forms part of the network of protected areas comprising the El Puuc State Biocultural Reserve, which includes several biocultural and conservation zones of peninsular importance (*e.g.,* Calakmul, Balam Kin, Balam Ku, Balam Kaax, Otoch Maax Yetel Koh, Sian Ka’an). The reserve contributes to a framework for the conservation of biocultural heritage, emphasizing both biological resource preservation and the maintenance of forest heterogeneity at the landscape scale. The use of the land is shaped by the region’s topography, which determines the distribution and suitability of soil types [33]. In the valleys, soils exhibit a clay accumulation horizon with moderate to high fertility, supporting a variety of agricultural uses, such as pastures and mechanized farming. On sloping areas, including hills, mounds, and knolls, soils contain a calcium carbonate (CaCO₃) horizon with high fertility, facilitating the exploitation of forest species (*e.g.,* mahogany, citrus) and the maintenance of forested reserve areas [34].

## Selection of sampling sites

To identify sampling sites, we conducted field visits and interviews with local peasants deeply rooted in the area, who provided critical insights into the location of areas with permanent vegetation and the successional stages of the forest. Based on this local knowledge, we identified three primary forest categories: (i) Mature Forest (BM), (ii) Mixed Forest in Development (MxFdev), and (iii) Early Forest with Agricultural Use (EarF/Agr).

**Fig 1:**
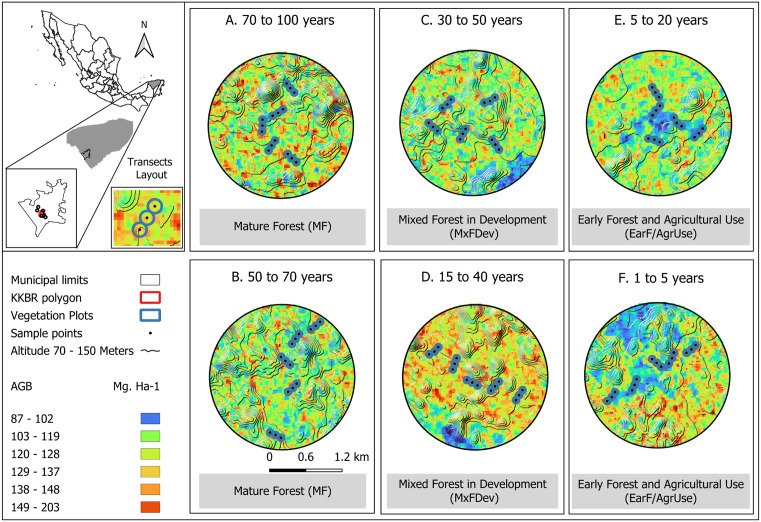
Study area in the Yucatan Peninsula, México. We show the 6 1.2-Km^2^ areas in each age class at the three successional stages. The black points represent the location of pitfall traps in each transect. The blue dots represent the vegetation plots. AGB: Aboveground Biomass (Mg. Ha-1). The base map was generated using QGIS software (http://www.qgis.org) and publicly available data on elevation, urban settlements, major roads, and national boundaries of Mexico, provided by the National Institute of Statistics and Geography (INEGI; http://www.inegi.org.mx/app/mapas/; accessed March 25, 2025). Terms of use are available at http://en.www.inegi.org.mx/inegi/terminos.html. Aboveground biomass were obtained through the forest biomass mapping using remote sensing data in R software v.4.0.1 (http://www.r-project.org) [37].

This phase of field recognition provided detailed information on the approximate age of use and management for each area, categorized into six successional stages: (A) Mature Forest: 70–100 years or more, (B) Mature Forest in Regeneration: 50–70 years, (C) Mixed Forest in Development: 30–50 years, (D) Early Forest: 15–40 years, (E) Early forest and agricultural use 5–20 years, and (F) Recent Agricultural Areas: recently cleared or actively cultivated lands (<5 year) (Fig 1). While there is some spatial heterogeneity in forest recovery time due to microenvironmental and management differences, all sites are structurally distinct and were validated using both field observations and previously published successional classifications for the region [35,36].

For each age class, we delimited a polygon with an approximate area of 1.2 km^2^, within which vegetation study plots were established. Within each polygon, five 100-m transects were distributed, each containing three pitfall traps spaced 25–30 m apart along the transect. To minimize spatial autocorrelation, transects were placed at least 250 m apart from one another and over 500 m from the polygon’s center.

In total, we established and characterized ninety plots throughout the chronological sequence, assessing both forest community structure (see below) and productivity across the identified successional stages.

### Dung beetle sampling

Dung beetles were sampled using pitfall traps. For each age class, five 100-m transects were established, with three pitfall traps per transect spaced 25–30 m apart, assigning one trap per plot [35]. In total, 90 pitfall traps were deployed across the forest chronosequence (Fig 1). Traps were baited with human dung and operated during two sampling periods: the cold–humid season (15 October–1 November 2021) and the warm–humid season (24 July–3 October 2022). Human dung represents an omnivore diet that allows capture a wider variety of species than herbivore dung and carrion favoring the sampling effort [36]. Each trap consisted of a 1 L container, filled three-quarters with 70% ethanol (v/v), and buried at ground level. A bait container (25 cm^3^) holding 40–50 g of human dung was suspended above the trap and covered with mesh to prevent direct contact with beetles. The traps were left in the field for 48 hours.

Captured individuals were processed in the Rural Ecology and Pathology Laboratory at Cinvestav-Mérida and preserved in 96% ethanol (v/v). Species identification was conducted using specialized taxonomic keys (Vaz-de-Mello et al. [38]; Edmonds & Zidek [39]; Howden & Young [40]), and determinations were verified against regional collections when necessary. All species were then assigned to, and body-size classes based on longitudinal axis measurements: large (L: 18–21.9 mm) and small (S: 3.1–17 mm). Functional groups included: Small Rollers Diurnals (SRD), Small Rollers Nocturnals (SRN), Small Tunnellers Diurnals (STD), Small Tunnellers Nocturnals (STN), Large Rollers Nocturnals (LRN), Large Tunnellers Diurnals (LTD), and Large Tunnellers Nocturnals (LTN), following the frameworks of Feer & Pincebourde [41] and Barragán et al. [6]. Biomass estimates were calculated by randomly selecting 5–30 individuals for species with moderate abundance and more than 90 individuals for hyper-abundant species in each age class. The number of individuals weighed was proportional to the total abundance of each species, ensuring representation across species with different capture frequencies. Selected individuals were dried at 50 °C for 48 hours and weighed using a precision balance. Total biomass for each species—including unmeasured individuals—was calculated by multiplying the mean dry weight of the selected individuals by the total number of captured individuals of that species in each age class.

### Microclimatic habitat conditions

Microclimatic conditions were monitored using HOBO ProV2 automatic sensors, with one sensor deployed per transect. In total, 30 microclimatic measurements were obtained across the chronosequence, as dataloggers were sequentially placed in the sampled transects and then relocated to subsequent forest age classes. Microclimatic conditions were monitored using HOBO ProV2 automatic sensors, deploying one sensor per transect for 10 consecutive days within each forest age class. After each sampling period, sensors were relocated sequentially to the following class, generating a total of 30 independent microclimatic records. Measurements were taken at 30-minute intervals. From these data, mean values (X ± SD) were calculated for each microclimatic variable, including minimum and maximum temperature (°C) and mean relative humidity (%). The total mean values obtained for each variable were used in the data analysis.

### Forest structure, diversity, and productivity

Vegetation structure for each forest age class was assessed by establishing a 12.6 m radius plot (500 m^2^) at each vegetation study plot, with each plot paired to a pitfall trap used for dung beetle sampling. Tree height was recorded by measuring the smallest and largest angles to the tree top from a minimum distance of 5 meters using a clinometer (SUUNTO PM-5/360 PC). Heights were then calculated with the trigonometric formula *h* = tan(*A*) x *d* + observer’s eye height, where *d* is the horizontal distance from the tree base and *A* is the measured angle to the tree top.

Diameter at breast height (DBH) was measured for all individuals with a diameter ≥2.5 cm at 1.3 m aboveground level. Canopy openness was quantified from photographs taken at a height of 2 m using a semi-spherical lens. The pixel values corresponding to areas without canopy cover were calculated using the Gap Light Analyzer (GLA) software, providing canopy openness values for each plot. All recorded individuals were identified to the species level “Table 1”.

**Table 1:** Characteristics (mean ± SD) of forest age classes corresponding to each successional stage of secondary tropical dry forest (SDF). Forest age and associated land-use activities were defined according to the multiple-use strategy practiced by Yucatecan Mayan families, as described in references [42,43]*.

Successsional Stage	Hunting pressure	Age Class (Years)*	Land Use*	Canopy Openness (%)	Temperature (°C)			Relative Humidity	Height Dominance Tree	Vegetal species Richness	Forest Productivity		
					Min	Max	Mean	RH (%)	HDTree (m)	*S*	Litter volume (m3. Ha-1)	AGB (Mg. Ha-1)	DBH (m2. Ha-1)
Mature Forest (MF)	Low	70 to 100 “*Suhuy K’ aax Noj Káax*”	Payment for environmental services	28.8 ± 9	22.9 ± 0.2	29.5 ± 0.4	26.±0.7	88.7	7.54 ± 0.2	29 ± 7	3028.2 ± 43.3	180.8 ± 13.5	6353.5 ± 220
		50 to 70 *Suhuy K’ aax Noj Káaxl*“	Payment for environmental services	31.1 ± 8	23.1 ± 0.2	30.1 ± 0.5	26.6 ± 0.3	87.8	7.43 ± 0.2	33.8 ± 8	3847.5 ± 111.3	143.2 ± 15.5	5470.3 ± 495
Mixed Forest in Development (MFReg, MxFDev)	Medium	30 to 50 “*Ka’ anal k’ aax*”	Beekeping, reforestation, hunting,	21.8 ± 3	23.4 ± 0.2	28.6 ± 0.4	26 ± 0.1	91.5	8.45 ± 0.1	42.2 ± 5	845.1 ± 26.9	211.8 ± 42.7	4157.3 ± 385
		15 to 40 “*Kelenche*”	Beekeping, reforestation, hunting,	25.1 ± 4	23.5 ± 0.3	28.6 ± 0.5	26 ± 0.1	88.2	8.22 ± 0.2	43 ± 8	1116.9.7 ± 24.5	229.8 ± 25.4	5559.3 ± 166
Early forest/ Agricultural and livestock use (EarF, AgrUse)	High	5 to 20 “*Ka’ anal-hubche*”	Plantations (pineapple, citrus, mango)	39.4 ± 10	21.5 ± 0.9	29.6 ± 0.4	25.6 ± 0.6	86.9	8.45 ± 0.2	23.4 ± 1	1647.7.7 ± 32.5	115.5 ± 71.1	3817.9 ± 585
		1 to 5 “*Hubche, Sak’ aab-hubche, Sak’aab*, Milpa”	Forest livestock, maize, cucurbit, bean	33.5 ± 21	23 ± 0.2	30.6 ± 0.9	26.8 ± 0.4	85.5	10.6 ± 4	23.4 ± 1	1303.6.4 ± 57.4	138.7 ± 74.7	2926.1 ± 428

We sampled five independent transects within each forest age class. Canopy Openness (CnpyOp), Relative Humidity % (RH), Height Dominance Tree (HDTree), Vegetal diversity richness (S), Litter volume (VLefLt), Aboveground Biomass (AGB), Diameter at Breast Height (DBH).

To characterize human influence on forest diversity and structural changes along the chronosequence, we calculated diversity indices for each forest age class and successional stage. These included the Shannon entropy index (H′) to assess species diversity and evenness, and Simpson’s diversity index (D), which emphasizes dominant species. We chose Simpson’s D rather than its inverse (1/D) because D directly quantifies dominance, which is ecologically informative in our context of early successional disturbance. Differences in vegetation community composition among age classes were assessed using the quantitative Jaccard similarity index (IJ), the Sørensen similarity index (SI), and the local contribution of beta diversity (LCBD) based on Whittaker’s beta diversity framework [44]. We included both Jaccard and Sørensen indices to capture both presence/absence-based and abundance-weighted community differences. The selection of diversity indices was guided by an assessment of their statistical performance—specifically, the distribution of Pearson correlation values and their skewness and leverage—to ensure robust and balanced comparisons across age classes. To further quantify variation in diversity patterns, we calculated inequality factors (IF₀,q) using Hill diversity numbers [45,46]. IF₀,₁ reflects uniformity derived from Shannon entropy, and IF₀,₂ reflects unevenness based on Simpson’s inverse concentration index. These factors were expressed as the ratio of diversity values (e.g., IF₀,q = D₀/ D₁,₂), allowing us to examine how inequality in diversity changes with succession.

### Forest productivity and habitat heterogeneity

Forest productivity and development across age classes were evaluated by estimating the dominant height, calculated as the average height of the tallest individuals among the 100 randomly selected trees measured in each independent area (transect) within each forest age class. Basal area and forest-use thresholds were calculated using allometric equations fitted for Yucatán dry forests, as described in [47,48], and incorporating the reported average density of woody species in the region [37]. Aboveground biomass (AGB) was estimated as the sum of the biomass of all individual trees (in kg) recorded in each transect, and then converted to megagrams per hectare (Mg ha ⁻ ¹). Additionally, leaf litter depth was measured as a proxy for productivity, using the average value from 48 measurements. These were obtained by taking four measurements within each of twelve 1 m^2^ quadrats randomly distributed across each plot. Leaf litter volume was estimated by calculating the volume of a cube (V = a^3^), where “a” corresponds to the measured depth in centimeters (Table 1).

### Data analysis

The completeness of the dung beetle community assemblages sampling was assessed using sensitivity values of species richness (^0^*D*), Shannon diversity (^1^*D*), and Simpson diversity (^2^*D*) with the iNEXT package [49]. A completeness index (*C*_*n*_) greater than 0.99 was obtained, indicating thorough sampling across all sites “S1 Table”.

To assess the influence of vegetation cover predictors (i.e., successional stages and age classes) on the diversity orders ^q^D of the dung beetle assemblages, a two-way ANOVA was performed. No transformation was applied to the ^q^D values. However, species abundance data used in predictive multivariate analyses were Hellinger-transformed, and logarithmic transformation (log₁₀) was applied to rank-dominance curves. Differences in dung beetle assemblage attributes—including species richness, abundance, and biomass—between study seasons were analyzed using a multiple analysis of variance (MANOVA), with significance evaluated through Games–Howell post hoc tests. The Games–Howell test was selected because it is appropriate when the assumption of homogeneity of variances is not met, and does not require equal sample sizes. To determine whether changes in the assemblages attributes between study seasons followed a normal distribution, the Shapiro–Wilk test was applied for univariate normality assessment. Data quality was further evaluated through confidence interval calculations (p < 0.05) and identification of outliers.

Changes in species assemblages composition among forest successional stages and age classes across seasons were analyzed using non-metric multidimensional scaling (NMDS). Additionally, range-dominance curves were constructed to describe assemblages composition and species dominance across forest improvement stages. Differences in the distribution of dung beetle diversity orders were evaluated using two-way ANOVA, with forest successional stage and age class as predictors of change. Bonferroni post-hoc tests were used to assess significance, and 95% confidence intervals were calculated. Bootstrapping was performed to estimate species richness, abundance, and biomass for the study seasons. To determine if there were differences in abundance and biomass across functional groups, one-way ANOVA was applied.

To reduce the number of variables and ensure their independence, a principal component analysis (PCA) was conducted using the vegan package (version 2.5–6) [50] on a matrix standardized to z-scores. Pearson correlation coefficients (*r*-values) were calculated to evaluate collinearity between variable pairs “S2 Fig”.The association between dung beetle species and vegetation attributes was analyzed using simple correspondence analysis (CA) based on species presence-absence, abundance, and biomass data. CA was selected because it is efficient for community species ordination using categorical or abundance data and is less sensitive to sampling error or gradient length. This makes it a robust and interpretable method compared to alternatives such as DCA, NMDS, or PCoA [51]. The influence of forest use intensity on dung beetle assemblage composition was evaluated using redundancy analysis (RDA) with the *OrdiR2step* function in the vegan package [52]. A Hellinger matrix was used for prior transformation of the species abundance data. Predictors of chronological forest succession were classified into three independent matrices: (i) environmental characteristics and habitat structure, (ii) forest diversity, and (iii) forest productivity. The criteria for assigning variables to each matrix were based on the conceptual understanding that forest cover in the region forms a mosaic of patches resulting from alternating cycles of agriculture and natural regrowth. These cycles are shaped by social, economic, and ecological drivers, where past land-use practices (e.g., burning during the agricultural phase) can exert long-term effects on subsequent vegetation development and ecosystem functioning during the regrowth phase [53,54]. The most significant variables in each matrix and their effects on dung beetle assemblage composition were identified using forward and backward stepwise selection procedures [55]. Variance partitioning was performed to separate the independent and joint effects of forest changes.

The spatial independence of forest improvement ages and dung beetle assemblages distribution was analyzed using distance-based redundancy analysis (*db*RDA) via the Capscale function. Adjusted *R^2^* values (adj. *R^2^*) were calculated, and variable significance was tested through iterative Monte Carlo permutations (999 permutations) [56]. To evaluate the influence of agroforestry systems on the ecological attributes of dung beetle assemblages (*e.g.,* ^q^*D* diversity orders, abundance, functional groups), multiple inference models were constructed using the Akaike Information Criterion for small samples (AICc) with the AICmodavg package [57]. The goodness-of-fit of plausible models (qAICc < 2) was determined by comparing the deviance of each selected model with that of a null model containing only the intercept. Candidate models were built using combinations of 13 predictor variables grouped into three matrices: environmental characteristics and habitat structure, forest diversity, and forest productivity [58]. All Statistical analyses were performed in R Studio version 4.2.2 [59].

## Results

### Dung beetle assemblages and guild composition

A total of 6,605 dung beetle individuals were recorded, representing 23 species and 13 genera “S1 Table”. The dissimilarity in dung beetle assemblage composition between successional stages and forest age classes in each season was 22.3% and 19.6%, respectively. These values, obtained from a Bray–Curtis dissimilarity analysis, represent the proportion of change in species composition across categories. This indicates that community structure is influenced by anthropogenic modification of forest cover and by seasonal rainfall patterns that regulate the abundance and activity of dung beetles (see S1 Fig).

**Fig 2:**
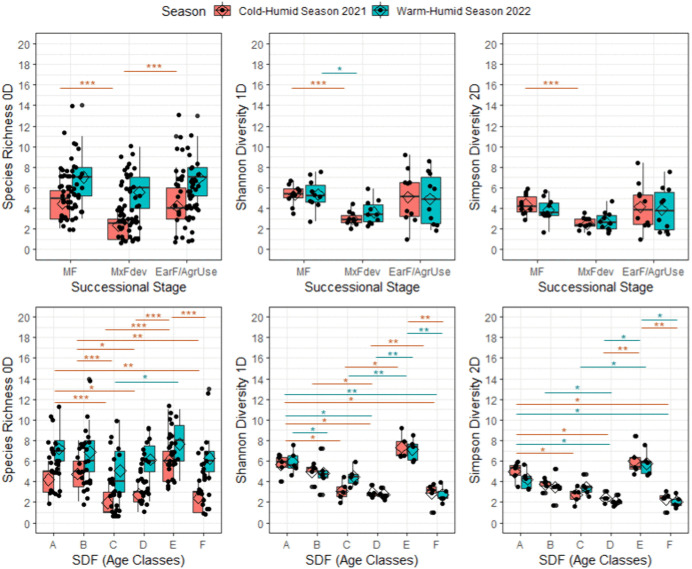
^q^*D* diversity orders of dung beetle assemblages captured in pitfall traps for each successional stage and Secondary Dry Forest (SDF) age classes in Southern Yucatan, Peninsula. MF: Mature Forest (A: 70–100 years; B. 50–70 years); MxFDev: Mixed Forest in Development (C. 30–50 years; D. 15–40 years); and EarF/ AgrUse: Early Forest and Agricultural Use (E. 5–20 years; F. 1–5 years). Species Richness ^0^*D*; Shannon Diversity ^1^*D*; Simpson Diversity ^2^*D*.

The warm–humid season of 2022 yielded higher diversity of dung beetles than the cold–humid season of 2021; however, the patterns of association between forest age classes or successional stages and the diversity orders (^*0*^*D,* ^*1*^*D,* ^*2*^*D*) were consistent between seasons (Fig 2). The two-way ANOVA revealed significant differences in frequent species diversity (^*1*^*D*) and dominant species diversity (^*2*^*D*) across both successional stages (^*1*^*D*: F(13,39) = 14.62, p < 0.001; ^*2*^*D*: F(8,415) = 7.47, p < 0.001) and age classes (^*1*^*D*: F(24,44) = 26.7, p = 0.001; ^*2*^*D*: F(20,857) = 18.5, p < 0.001). Species richness (^*0*^*D*) did not show significant differences across forest age classes (^*0*^*D*: F(2,442) = 8.467, p = 0.08) but did differ significantly across successional stages (^*0*^*D*: F(5,663) = 19.6, p = 0.009).

Overall, diversity values were higher in older forests and lower in agricultural areas, except for the 5–20-year age class (Early Forest and Agricultural Use), which showed unexpectedly high diversity. This pattern was most pronounced for frequent species diversity (^*1*^*D*) but was evident across all diversity metrics (^*0*^*D,* ^*1*^*D,* ^*2*^*D*). The higher values of species richness and diversity in the 5–20-year class were linked to the presence of open-habitat dung beetle species such as *Canthon indigaceus chevrolati* (Harold, 1868), Sisyphus mexicanus (Harold, 1863), *Malagoniella astyanax yucateca* (Harold, 1863), *Pseudocanthon perplexus* (LeConte, 1847), and *Copris lugubris* (Boheman, 1858). These species contributed to a marked increase in diversity within successional stages associated with agricultural use and vegetation regrowth between 5 and 20 years (Fig 2).

**Fig 3:**
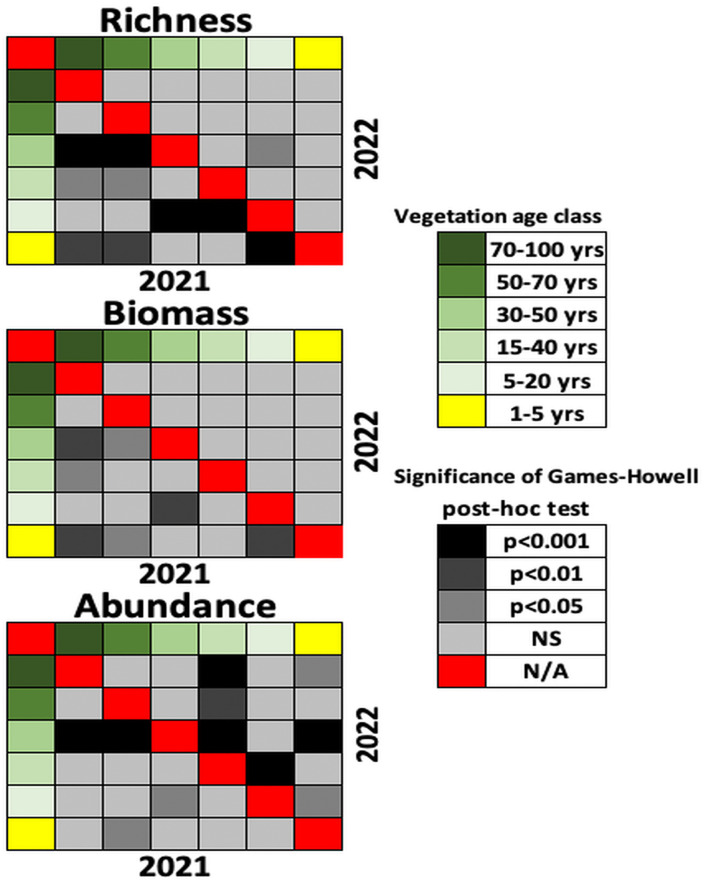
Pairwise differences in species richness, biomass, and abundance across vegetation age classes in two sampling seasons. Matrix of significance levels from post-hoc comparisons derived from MANOVA analyses. Lower triangle (below the diagonal) shows pairwise differences for the 2021 cold-humid season; upper triangle (above the diagonal) shows pairwise differences for the 2022 warm-humid season. Colors indicate significance levels based on p-values. Vegetation age classes range from mature forest (70–100 years) to agricultural use (1–5 years). Detailed MANOVA results for each response variable are provided in S2 (richness), S3 (biomass), and S4 Tables (abundance).

Species richness was consistently higher in the 2022 warm-humid season across all vegetation classes compared to the 2021 cold-humid season. In contrast, abundance and biomass showed less variation between years, although abundance increased in 2022 in several sites, particularly those under agricultural use. These patterns are supported by data in S1 Table, which details species-level abundance by successional stage, age class, and year. The most pronounced differences in species richness and biomass during the 2021 cold-humid season were observed between mature forests (70–100 years) and all younger forest categories, including mixed forests, recently recovered areas (5–20 years), and the agricultural site (1–5 years of recovery). In terms of abundance, significant contrasts were also found between mature forests and both mixed forests and the agricultural site, as well as between young mixed forests (15–40 years) and the agricultural site. In contrast, during the 2022 warm-humid season, significant differences between sites were detected only for dung beetle abundance, with the strongest differences occurring between mature forests and young mixed forests. These results are illustrated in Fig 3, which shows pairwise comparisons within each year, and are supported by the MANOVA results detailed in S2 (species richness), S3 (biomass), and S4 Tables (abundance).

**Fig 4:**
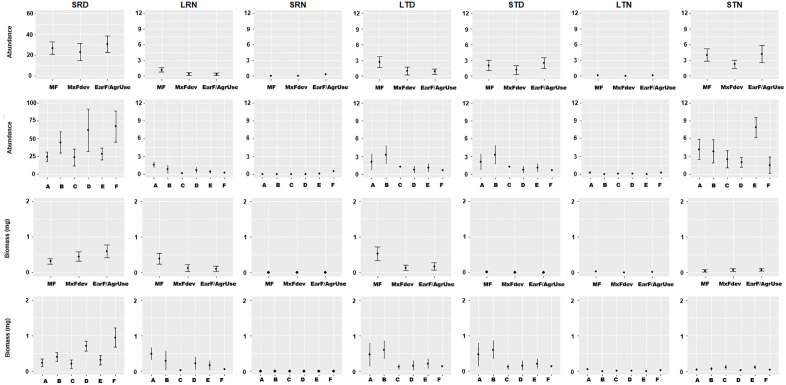
Guild structure of functional groups in the dung beetle assemblages for abundance and biomass (±SE) for each successional stage and Secondary Dry Forest (SDF) age classes. *Abbreviations*: small rollers diurnal (SRD), large rollers nocturnal (LRN), small rollers nocturnal (SRN), large tunnellers diurnal (LTD), small tunnellers diurnal (STD), large tunnellers nocturnal (LTN), small tunnellers nocturnal (STN). MF: Mature Forest (A: 70 to 100 years; B. 50 to 70 years); MxFDev: Mixed Forest in Development (C. 30 to 50 years; D. 15 to 40 years; and EarF/AgrUse: Early Forest and Agricultural Use (E. 5 to 20 years; F. 1 to 5 years).

Both abundance and biomass were sensitive attributes for assessing responses to land-use change. Small diurnal rollers (SRD) dominated across the forest chronosequence, with both abundance and biomass showing a progressive increase under greater land-use intensity. In contrast, small nocturnal tunnellers (STN) exhibited a decline in abundance at intermediate levels of recovery (*i.e.,* mixed forest in development), being more abundant in early forest/plantations. Large-sized functional groups, including large nocturnal rollers (LRN) and large diurnal tunnellers (LTD), displayed decreasing trends in both abundance and biomass throughout the chronosequence, with the steepest declines observed in areas under intensive agricultural use. This trend was also showed by small diurnal tunnellers (STD) only in response to forest age “Fig 4”.

**Fig 5:**
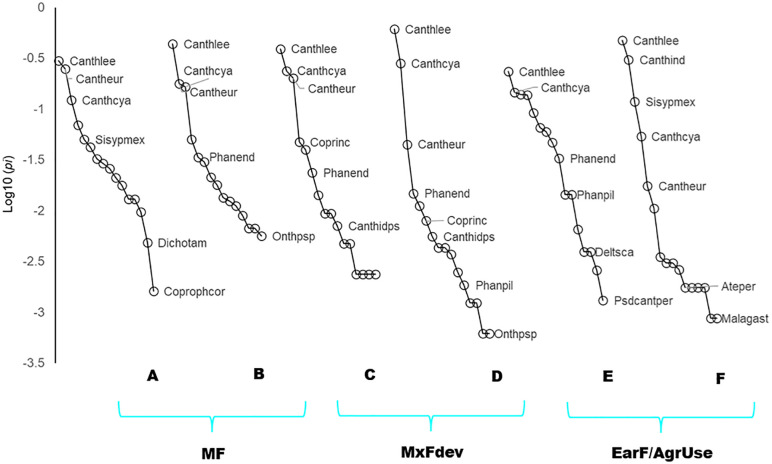
Distributional dominance curves of the dung beetle assemblage species for each age class of Secondary Dry Forest (SDF) in the successional stages. Species codes and full names are provided in S1 Table.

Community structure analyses revealed that *Canthon leechi* (Martínez, Halffter & Halffter, 1964) was the numerically dominant species, accounting for 45% of the sampled individuals. This was followed by *Canthon cyanellus* (LeConte, 1859) (16%) and *Canthon euryscellis* (Bates, 1887) (9%), which were prevalent across most forest age classes. However, in areas with recent and intensive agricultural exploitation (1–5 years) and in forests adjacent to pastures and livestock systems, the assemblage structure was notably modified by the presence of *C. indigaceus chevrolati* (Harold, 1868), which contributed 10% of the individuals. Additionally, *Sisyphus mexicanus* (Harold, 1863) exhibited a gradual increase in abundance in the youngest forest age classes, particularly in early forests (<20 years) and agricultural areas subjected to continuous use (1–5 years of intensive activities), comprising 6% of the total individuals recorded in these areas “Fig 5”.

### Dung beetle assemblage distribution and influences of forest attributes

The correspondence analysis (CA) described the distribution patterns of dung beetle species along the forest chronosequence and identified their associations with forest-use attributes, based on the cumulative contributions of CA1 and CA2. The analysis explained 37.4% of the variation in species community distribution (CA1: Height Dominance of Trees – “HDTree”; CA2: Simpson Vegetation Index – “D”), 55.3% of the biomass (CA1: Inequality Factor based on Shannon Exponential Diversity – “IF0.1”; CA2: Canopy Openness – “CnpyOp”), and 67.1% of the abundance (CA1: Aboveground Biomass – “AGB”; CA2: Height Dominance of Trees – “HDTree”) (S3 Fig).

Changes in species richness, biomass, and abundance across forest age classes were primarily influenced by interactions between forest productivity predictors (DBH, VLefLt, AGB) and forest diversity metrics (IF₀,₁, IF₀,₂, H′, D, LCBD, IJ). Species richness showed a positive association with aboveground biomass (AGB) and the exponential Shannon inequality factor (IF₀,₁), but a negative association with litter volume (VLefLt) and the inequality factor based on the inverse Simpson concentration index (IF₀,₂). Biomass was positively influenced by both IF₀,₁ and VLefLt, while abundance was positively associated with AGB, VLefLt, and IF₀,₂. In addition, environmental variables related to vegetation structure and microclimatic conditions (HDTree, CnpyOp, T°Max, %RH) interacted with forest diversity metrics to further explain the observed variation. Canopy openness (CnpyOp) exhibited variable effects on dung beetle assemblage attributes, showing a positive influence only on abundance (Table 2).

**Table 2:** Variance partitioning and variable selection using redundancy analysis (RDA) and distance-based redundancy analysis (dbRDA) showing the unique and shared contributions of three sets of explanatory variables to variation in dung beetle assemblages. [AUB], [BUC], and [AUC] represent the variation fractions explained jointly or uniquely by combinations of predictor sets: environmental variables (A), forest productivity (B), and forest diversity metrics (C). See methods for detailed matrix definitions.

Community Structure	Residual Variance	Variance Explained	A. Enviromental characteristics and habitat structure	B. Forest productivity	C. Forest diversity	Variation Partitioning Fractions		
						[AUB]	[BUC]	[AUC]
Species Richness	0.804	0.206	0.004	0.034	0.091	0.038	0.125	0.095
Biomass	0.711	0.289	0.027	0.127	0.11	0.154	0.237	0.38
Abundance	0.571	0.429	0.06	0.151	0.119	0.249	0.27	0.179
**Community Structure**	**Selection procedure**	**Global *R***^**2**^ **Adjust (RDA All vars.)**	***R***^**2**^ **Adjust (RDA Selected vars.)**	**Model ANOVA**			***db*RDA1**	***db*RDA2**
				***Df***	***F***	***p-value***	**Variance Explained (%)**	**Variance Explained (%)**
Species Richness	Backward	0.143	0.191	8	1.96	< 0.001	13.06	7.8
Biomass	Backward	0.284	0.317	11	1.83	< 0.001	14.25	9.3
Abundance	Forward	0.471	0.487	10	2.56	< 0.001	24.5	7.09

Acronyms for predictors: IF₀,₁: Shannon exponential diversity; IF₀,₂: Simpson inverse concentration; H’: Shannon entropy; D: Simpson diversity; LCBD: Local contribution to beta diversity; IJ: Jaccard index; DBH: Diameter at breast height; VLefLt: Leaf litter volume; AGB: Aboveground biomass; HDTree: Tree height dominance; CnpyOp: Canopy openness; T° Max: Maximum temperature; RH: Relative humidity.

Table 2. Variance partitioning and variable selection using redundancy analysis RDA and distance-based redundancy *db*RDA of the set of variables grouping in matrices that describe the influence of agroforestry system attributes in explaining changes in the dung beetle assemblages.

### Influence of forest changes on dung beetle assemblage attributes

**Fig 6:**
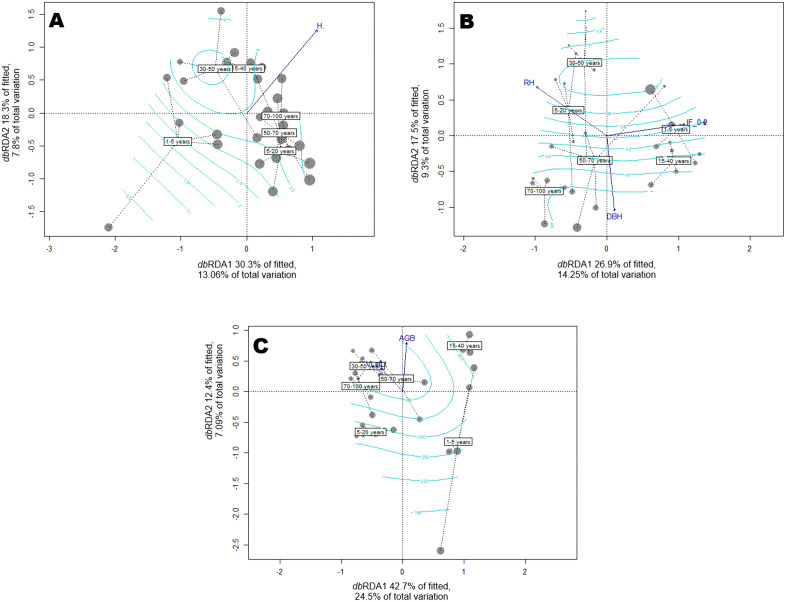
Dung betlee assemblages composition biplot from distance-based redundancy analysis *db*RDA for changes of dung beetle assemblages attributes across ages forest classes: A. Species richness; B. Biomass; and C. Abundance. Arrows represent the restricted variables. Contour lines correspond to the explanatory variable representing the gradient of change in forest chronosequence. *H’*: Shannon entropy index; RH: Relative Humidity (%); DBH: Diameter at Breast Height; *IF*_0.2_: Inequality Factor based in unevenness Simpson’s inverse concentration; VLefLt: Leaf litter Volume; AGB: Aboveground Biomass.

Species richness was primarily influenced by high plant diversity, as described by the Shannon entropy index (*H*′) (adj. *R^2^* = 0.19; *p* < 0.001; explained variance = 20.8%) “Fig 6A”. Dung beetle biomass was influenced by changes in forest diversity (*IF*₀,₂), basal area (DBH), and relative humidity (% RH) (adj. *R^2^* = 0.31; *p* = 0.05; explained variance = 23.51%) “Fig 6B”. Changes in abundance were influenced by variables describing standing forest productivity corresponding to aboveground biomass (AGB) and leaf litter volume (VLefLt) (*adj. R^2^* = 0.48; *p* = 0.001; explained variance = 31.6%) “Fig 6C”. This analysis highlights the strong influence of forest productivity, diversity, and microclimatic factors on dung beetle community structure, emphasizing their sensitivity to forest-use patterns and recovery stages.

### Biodiversity responses to successional patterns

The attributes of the dung beetle assemblages showed distinct responses to models describing changes within the agroforestry system. Species richness (^0^*D*) was positively associated with leaf litter volume, which emerged as the strongest predictor in the model set. In contrast, frequent species diversity (^1^*D*) and dominant species diversity (^2^*D*) were positively associated with canopy density, but both diversity orders exhibited negative responses to changes in forest community evenness (Table 3). Abundance and biomass showed consistent and positive responses to predictors describing changes in plant uniformity, specifically the diversity inequality factors (*IF*₀,₁ and *IF*₀,₂) “Table 3”.

**Table 3:** Models predicting changes in ecological attributes and functional groups of the dung beetle assemblages along the forest system, assessed using the Akaike information criterion corrected for small sample size (AICc). Estimated parameters (No), including model mean (*ϐ*) and standard error (SE), intercept (k), AICc, difference in AICc (ΔAICc), AICc weight (*Wi*), adjusted *R*^2^, and evidence ratio (ER) (i.e., Akaike weight of the top-ranked model/Akaike weight of the second-best ranked model).

Model	Media	SE	k	AIC	Δ AICc	Wi	R^2^		ER
^0^ *D*									
AGB	0.003	0.004	3	10.65	4.85	0.07	8.51		11.28
VLefLt	−0.00001	0.000005391	3	5.8	0	0.76	59.21	.	11.28
*IF*_0.1_	0.14	0.29	3	10.84	5.03	0.06	5.63		
*IF*_0.2_	0.02	0.82	3	11.08	5.28	0.05	1.69		
CnpyD	−0.01	0.03	3	11	5.2	0.06	2.98		
^1^ *D*									
AGB	−0.03	0.02	3	30.72	0.82	0.2	29.84		
VLefLt	0.00004	0.000044	3	31.12	1.86	0.12	16.49		
*IF*_0.1_	−1.45	1.54	3	30.99	1.74	0.13	18.21		
*IF*_0.2_	−0.63	0.44	3	29.73	0.48	0.24	33.68		1.27
CnpyD	0.25	0.16	3	29.25	0	0.31	38.78		1.27
^2^ *D*									
AGB	−0.02	0.02	3	27.2	1.43	0.22	33.49		2.05
VLefLt	0.000018	0.000038	3	29.32	3.56	0.08	5.27		
*IF*_0.1_	−0.89	1.3	3	28.99	3.22	0.09	10.45		
*IF*_0.2_	−0.44	0.38	3	27.92	2.15	0.16	25.07		
CnpyD	0.22	0.12	3	25.77	0	0.45	47.63		2.05
Abundance									
AGB	−1.53	7.85	3	100.65	14.19	0	0.94		
VLefLt	−0.01	0.01	3	100.14	13.69	0	8.91		
*IF*_0.1_	948	197	3	89.23	2.78	0.2	85.23	**	4.01
*IF*_0.2_	311.49	49.87	3	86.45	0	0.8	90.7	**	4.01
CnpyD	5.08	59.75	3	100.69	14.24	0.001	0.18		
Biomass									
AGB	−0.07	0.09	3	46.82	6.48	0.02	13.04		
VLefLt	0.00009	0.0002	3	47.3	6.95	0.02	5.87		
*IF*_0.1_	9.82	3.75	3	41.66	1.32	0.32	63.2	.	1.93
*IF*_0.2_	3.3	1.07	3	40.34	0	0.62	70.46	*	1.93
CnpyD	0.2	0.71	3	47.54	7.19	0.02	2.03		
SRD									
AGB	−0.33	8.33	3	101.36	13.91	0.001	0.04		
VLefLt	−0.01	0.01	3	100.63	13.18	0.001	11.56		
*IF*_0.1_	983.9	228.5	3	90.99	3.54	0.15	82.26	*	5.86
*IF*_0.2_	328.07	54.18	3	87.45	0	0.85	90.16	**	5.86
CnpyD	−6.11	63.11	3	101.35	13.9	0.001	0.23		
LRN									
AGB	0.02	0.09	3	46.75	3.84	0.08	0.89	.	
VLefLt	0.0002	0.0001	3	42.91	0	0.58	47.72		4.47
*IF*_0.1_	−4.11	5.37	3	45.98	3.07	0.12	12.78		
*IF*_0.2_	−1.36	1.7	3	45.9	3	0.13	13.85		4.47
CnpyD	−0.01	0.67	3	46.8	3.89	0.08	0.01		
SRN									
AGB	−0.03	0.03	3	33.1	13.7	0.001	18.79		
VLefLt	−3.4E-05	0.0001	3	33.8	14.4	0.001	8.73		
*IF*_0.1_	3.89	0.58	3	19.4	0	0.78	91.72	**	3.52
*IF*_0.2_	1.21	0.23	3	21.9	2.52	0.22	87.4	**	3.52
CnpyD	0.1	0.23	3	34	14.65	0.001	4.86		
LTD									
AGB	−0.07	0.16	3	54.12	11.41	0.003	4.42		
VLefLt	0.001	0.0001	3	42.71	0	0.98	85.73	**	129.96
*IF*_0.1_	−11.39	9.2	3	52.44	9.73	0.01	27.72		129.96
*IF*_0.2_	−3.12	3.07	3	53.01	10.31	0.01	20.48		
CnpyD	0.25	1.25	3	53.33	11.62	0.003	0.95		
STD									
AGB	−0.34	0.20	3	56.54	2.41	0.13	42.33		
VLefLt	0.001	0.0003	3	54.14	0	0.44	61.38	**.**	1.21
*IF*_0.1_	−7.46	16.64	3	59.55	5.14	0.03	4.79		
*IF*_0.2_	−3.28	5.18	3	59.28	5.41	0.03	9.09		
CnpyD	3.05	1.28	3	54.53	0.39	0.36	58.8	**.**	1.21
LTN									
AGB	0.002	0.02	3	29.38	1.59	0.13	0.23		
VLefLt	−2.5E-06	0.00004	3	29.39	1.6	0.13	0.1		
*IF*_0.1_	1.31	1.18	3	27.79	0	0.3	23.45		1.05
*IF*_0.2_	0.4	0.38	3	27.89	0.1	0.28	22.15		1.05
CnpyD	−0.07	0.15	3	29.1	1.31	0.15	4.77		
STN									
AGB	−0.78	0.5	3	67.67	3.81	0.12	37.32		6.72
VLefLt	0.0003	0.001	3	70.41	6.54	0.03	1.12		
*IF*_0.1_	−18.12	40.35	3	70.18	6.32	0.03	4.8		
*IF*_0.2_	−10.44	12.09	3	69.45	5.59	0.05	15.72		
CnpyD	7.87	2.78	3	63.86	0	0.77	66.78	*	6.72

CnpyOp: Canopy Openness; VLefLt: Litter volume; AGB: Aboveground Biomass; *IF*₀,₁: standard forest diversity uniformity value based on entropy Shannon exponential; *IF*₀,₂: unevenness forest diversity measure based on Simpson inverse concentration. *Abbreviations*: Species Richness (^0^*D*), Shannon Diversity (^1^*D*), Simpson Diversity (^2^*D*), small rollers diurnal (SRD), large rollers nocturnal (LRN), small rollers nocturnal (SRN), large tunnellers diurnal (LTD), small tunnellers diurnal (STD), large tunnellers nocturnal (LTN), small tunnellers nocturnal (STN). Significance level: 0‘***’, 0.001‘**’, 0.01‘*’, 0.05 ‘.’.

### Functional group responses to agroforestry system productivity and diversity

The abundance of large rollers (LRN) was primarily explained by agroforestry system productivity variables, accounting for 59% of the deviance explained, while large tunnellers (LTD) showed an even stronger association, with 86% of their deviance explained by the same predictor set. Small diurnal rollers (SRD) abundance was positively associated with plant diversity changes, particularly with the inequality factors ***IF*₀,_₁_** and ***IF*_₀_,₂**, reflecting responses to diversity patterns across the agroforestry system. Species from small nocturnal (STN) and small diurnal (STD) tunneller groups, typically associated with closed habitats, showed consistent and positive associations with canopy density and leaf litter volume “Table 3”.

## Discussion

Our original hypothesis proposed that dung beetle community attributes—namely diversity patterns and functional group composition—would respond to forest succession in distinctive ways, influenced by the degree of habitat modification through changes in environmental conditions, vegetation structure, forest diversity, and productivity. This perspective allowed us to examine the effects of the natural recovery trajectory of tropical dry forest under a specific human management system in the southern Yucatán Peninsula. We found a close association between the ecological and functional attributes of dung beetle assemblages and the environmental changes that shape habitat conditions and ecosystem functional dynamics [10]. This was reflected in a stepwise response of ^q^*D* diversity patterns, driven by forest cover predictors that account for decadal and seasonal habitat fluctuations. These patterns also enabled us to disentangle the effects of historical land-use intensity and temporal landscape configuration on community assembly processes. We observed clear shifts in dung beetle functional traits—including body size, diel activity, and dung relocation strategy (the latter inferred based on sexual dimorphism) —associated with increasing land-use intensification along the forest chronosequence. These findings offer insight into key ecological processes underlying biodiversity and ecosystem functioning (*e.g.,* nutrient recycling), as inferred from well-established dung beetle attributes, within a socio-ecological context shaped by sustainable management practices and local ecological knowledge of a disturbance-sensitive tropical forest system [60].

### Dung beetle assemblage patterns and responses over the course of succession

Changes in ^q^*D* diversity orders for dung beetle assemblages showed significant differences across successional stages and forest age classes. Notably, our findings parallel those observed in fragmented rainforest systems in southeastern Brazil, where longitudinal studies over five years on dung beetle communities revealed functional group-specific thresholds in community dynamics [61]—a pattern mirrored in our results for 0D species richness and the abundance of small diurnal rollers (SRD). These trends suggest that observed shifts in species richness and functional dominance along the chronosequence may be strongly influenced by life history traits such as body size and nesting behavior, as well as by their ecological relationships with soil properties that govern species distribution [62,63]. For instance, we found that the abundance of large-bodied nocturnal rollers (LRN) and large tunnellers (LTD) was strongly associated with higher levels of aboveground biomass and leaf litter volume, which are proxies of soil organic matter and microhabitat complexity—factors known to influence nesting suitability and resource availability for these functional groups.

Moreover, historical land-use legacies and traditional management practices in the Maya Lowlands have produced a domesticated forest landscape that continues to shape species composition and ecological functions [64]. Thus, while dung beetle assemblages clearly respond to successional gradients, their recovery appears to be mediated by broader ecological and anthropogenic drivers, including disturbance intensity and restoration timelines [19]. This complexity helps explain why species assemblage dissimilarity between successional stages and age classes remains relatively consistent with forest change predictors at our study sites.

These patterns may also reflect feedback processes among ecological communities, where shifts in one group—such as plants or mycorrhizae—can cascade into varied responses in other groups, including replacement, persistence, or stasis, ultimately influencing the trajectory of community change [65]. Notably, our findings diverge from those reported in a tropical dry forest in Brazil under a slash-and-burn system, where dung beetle diversity remained relatively stable across a 4–50-year recovery gradient [66]. This contrast underscores the importance of regional context, land-use history, and the type of management system in shaping biodiversity outcomes during ecological succession.

### The diversity–disturbance relationship: biodiversity changes and ecological processes in predicting ecosystem trajectories

The threshold-step feedback patterns observed in dung beetle community diversity and assemblage composition across successional stages and forest age classes suggest that community structure and the distribution of functional groups are shaped by ecological filters imposed by human disturbance. These filters likely operate through mechanisms of niche partitioning, which allow certain species to dominate under specific conditions of productivity and environmental filtering [67]. Traits such as large body size, nocturnal or diurnal activity, nesting behavior (roller vs. tunneller), and colonization speed influence how species respond to soil conditions and resource availability across the chronosequence [62,63]. In this context, land-use management further selects for functional traits that enhance persistence in disturbed environments—favoring species with rapid dispersal, tolerance to microclimatic variation, or generalist feeding and nesting strategies [68].

These processes lead to measurable shifts in the proportional representation of functional groups, which are closely aligned with patterns of ecosystem use—especially mammalian activity. The responses of these groups serve as valuable indicators of ecological integrity, as changes in species abundance and biomass across successional gradients reflect broader trends of ecological degradation or recovery. This supports the concept of biodiversity–ecosystem function coupling, where community-level shifts follow a redundant–compensatory dynamic: despite species turnover, functional overlap may sustain ecosystem processes [69–71]. In our study, this was evidenced by the compensatory relationship between functional groups across the forest chronosequence. Specifically, in older successional stages where large-bodied nocturnal tunnelers (LNT)—typically associated with high biomass burial rates—became less abundant, their functional role appeared to be partially maintained by the increased dominance of small diurnal rollers (SRD), which also contribute to dung removal and nutrient cycling. This shift illustrates how different species with overlapping functions may stabilize key ecosystem processes under changing habitat conditions.

Temporal variation in dung beetle ecological attributes and functional traits across successional stages illustrates how biodiversity shifts transcend site boundaries and influence key ecological processes. For example, in our study, early successional sites showed reduced biomass and species richness but were dominated by small diurnal rollers, whereas more mature sites supported a higher abundance and biomass of large diurnal tunnelers. These changes are likely tied to nutrient cycling dynamics, as both body size and biomass are known to modulate dung burial rates and soil aeration. Thus, land-use regimes indirectly shape these processes by filtering for species with specific functional traits [72,73].

A prime example is the role of plant litter inputs in ecosystem function, acting as essential subsidies in both terrestrial and aquatic systems. In our study, we found that dung beetle biomass and functional dominance were strongly associated with successional gradients in forest age and productivity. For instance, the biomass and abundance of large-bodied rollers were highest in the oldest forest, where resource availability and microclimatic stability likely support their persistence and functional role in nutrient cycling. These processes are further influenced by plant litter quality and quantity, shaped by species richness and phenological variation, which drive detritivore activity and organic carbon turnover. Understanding these interactions in the context of land-use change and forest succession is crucial for forecasting long-term ecosystem trajectories and guiding effective, locally grounded conservation strategies [74–77].

### Implications for conservation and policies

Our findings suggest that land-use change under the traditional milpa harvesting system supports the natural recovery of tropical dry forests in the southern Yucatán Peninsula. This system enhances a mosaic of habitats across successional stages, as evidenced by shifts in species composition, abundance, and functional group structure within dung beetle assemblages. These results are consistent with previous research highlighting the regenerative role of traditional milpa practices in fostering forest heterogeneity and soil health across temporal scales [26,27].

This evidence reinforces the potential of locally adapted, low-intensity land-use regimes to sustain both biodiversity and ecosystem functioning, even within landscapes subject to human disturbance. Moreover, recognizing that such systems have evolved through a deep historical relationship between people and forests adds important context for interpreting patterns of biodiversity and ecological resilience in these regions [78].

Accordingly, we advocate for a conservation approach that promotes the active participation of local stakeholders, in collaboration with conservation authorities. This strategy would not only strengthen the governance of protected areas but also support the identification and stewardship of human-managed landscapes that function as transit habitats or biological corridors for wildlife. Emphasizing the role of traditionally managed systems like milpa within conservation frameworks could enhance both biodiversity outcomes and community resilience in tropical dry forest regions [79,80].

## Supporting information

S1 Table Species abundance of dung beetles collected in each successional stage of secondary dry forest (SDF) in the southern Yucatan Peninsula, México, for both sampled years (2021 and 2022). FG: Functional Groups; MF: Mature Forest, MFReg: Mature Forest in Regeneration, MxFDev: Mixed Forest in Development, EarF: Early Forest, AgrUse: Agricultural Use.
(DOCX)

S2 Table Multivariate analysis of variance (MANOVA) results for dung beetle species richness (q0) across forest successional stages and age classes in the southern Yucatan Peninsula, Mexico. (DOCX)

S3 Table Multivariate analysis of variance (MANOVA) results for biomass of dung beetle assemblages across successional stages and forest age classes of secondary dry forest (SDF) in the southern Yucatán Peninsula, Mexico. (DOCX)

S4 Table Multivariate analysis of variance (MANOVA) results for total abundance of dung beetles collected across successional stages and age classes of secondary dry forest (SDF) in the southern Yucatán Peninsula, México, during the 2021 and 2022 sampling seasons. (DOCX)

S1 Fig A. Non-metric multidimensional ordination (NMDS) of community assemblages and dissimilarity percent between seasons based on species richness. (TIFF)

S2 Fig Pearson correlation matrix for the set of variables describing changes in the forest system. (TIFF)

S3 Fig Simple correspondence analysis CA for the species of the dung beetle community: A. Species of the community; B. Total biomass of each species; C. Total abundance of each species. The vectors represent the direction of the most important variables of the forest in the chronosequence. *D*: Simpson’s diversity index; HDTree: Dominance tree height; CnpyOp: Canopy openness; *IF*_0,1_: Shannon exponential entropy-based inequality factor; AGB: Aboveground biomass.
(JPG)
