# Supplementary material for: Dung beetle assemblage changes along a chronosequence in a recovering tropical dry forest
Source: PLoS One. 2025 Dec 4;20(12):e0337635. doi: 10.1371/journal.pone.0337635 (PMC12677776; doi:10.1371/journal.pone.0337635)
Supplement: S1 Table — FG: Functional Groups; MF: Mature Forest, MFReg: Mature Forest in Regeneration, MxFDev: Mixed Forest in Development, EarF: Early Forest, AgrUse: Agricultural Use. (DOCX) [file pone.0337635.s001.docx]

S1 Table. Species abundance of dung beetles collected in each successional stage of secondary dry forest (SDF) in the southern Yucatan Peninsula, México, for both sampled years (2021 and 2022). FG: Functional Groups; MF: Mature Forest, MFReg: Mature Forest in Regeneration, MxFDev: Mixed Forest in Development, EarF: Early Forest, AgrUse: Agricultural Use.
