# Supplementary material for: Dung beetle assemblage changes along a chronosequence in a recovering tropical dry forest
Source: PLoS One. 2025 Dec 4;20(12):e0337635. doi: 10.1371/journal.pone.0337635 (PMC12677776; doi:10.1371/journal.pone.0337635)
Supplement: S2 Table — (DOCX) [file pone.0337635.s002.docx]

**S2 Table. Multivariate analysis of variance (MANOVA) results for dung beetle species richness (q0) across forest successional stages and age classes in the southern Yucatan Peninsula, Mexico.**
