# Supplementary material for: Dung beetle assemblage changes along a chronosequence in a recovering tropical dry forest
Source: PLoS One. 2025 Dec 4;20(12):e0337635. doi: 10.1371/journal.pone.0337635 (PMC12677776; doi:10.1371/journal.pone.0337635)
Supplement: S3 Table — (DOCX) [file pone.0337635.s003.docx]

**S3 Table. Multivariate analysis of variance (MANOVA) results for aboveground biomass of dung beetle assemblages across successional stages and forest age classes of secondary dry forest (SDF) in the southern Yucatán Peninsula, Mexico.**
