# Supplementary material for: Dung beetle assemblage changes along a chronosequence in a recovering tropical dry forest
Source: PLoS One. 2025 Dec 4;20(12):e0337635. doi: 10.1371/journal.pone.0337635 (PMC12677776; doi:10.1371/journal.pone.0337635)
Supplement: S4 Table — (DOCX) [file pone.0337635.s004.docx]

**S4 Table. Multivariate analysis of variance (MANOVA) results for total abundance of dung beetles collected across successional stages and age classes of secondary dry forest (SDF) in the southern Yucatán Peninsula, México, during the 2021 and 2022 sampling seasons.**
