# Supplementary figures and images for: Dung beetle assemblage changes along a chronosequence in a recovering tropical dry forest
Source: PLoS One. 2025 Dec 4;20(12):e0337635. doi: 10.1371/journal.pone.0337635 (PMC12677776; doi:10.1371/journal.pone.0337635)

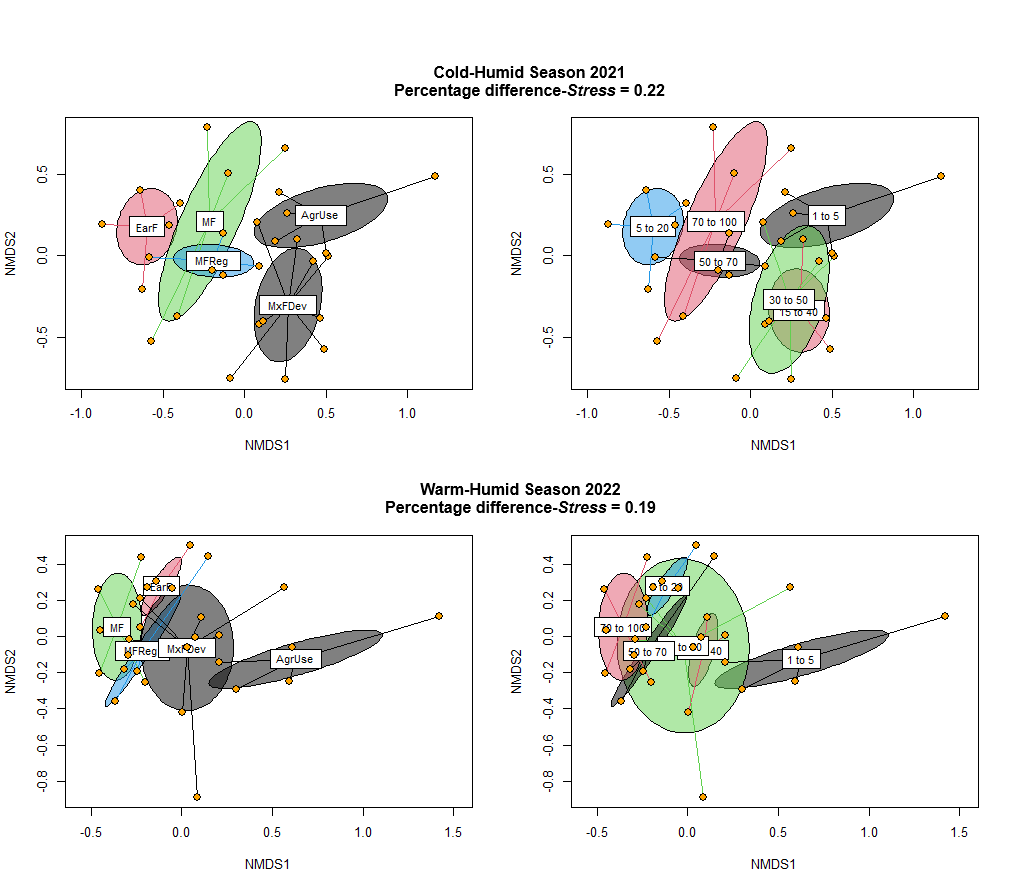

Supplement: S1 Fig — (TIFF) [file pone.0337635.s005.tiff]

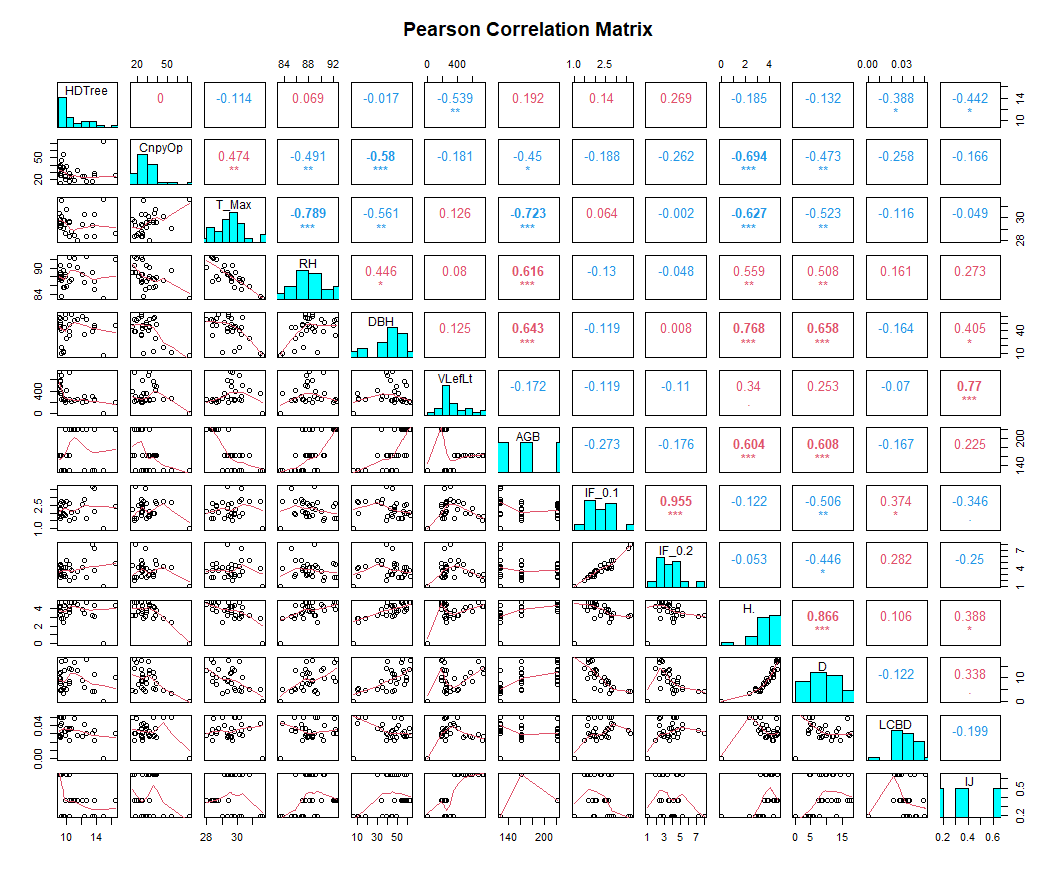

Supplement: S2 Fig — (TIFF) [file pone.0337635.s006.tiff]

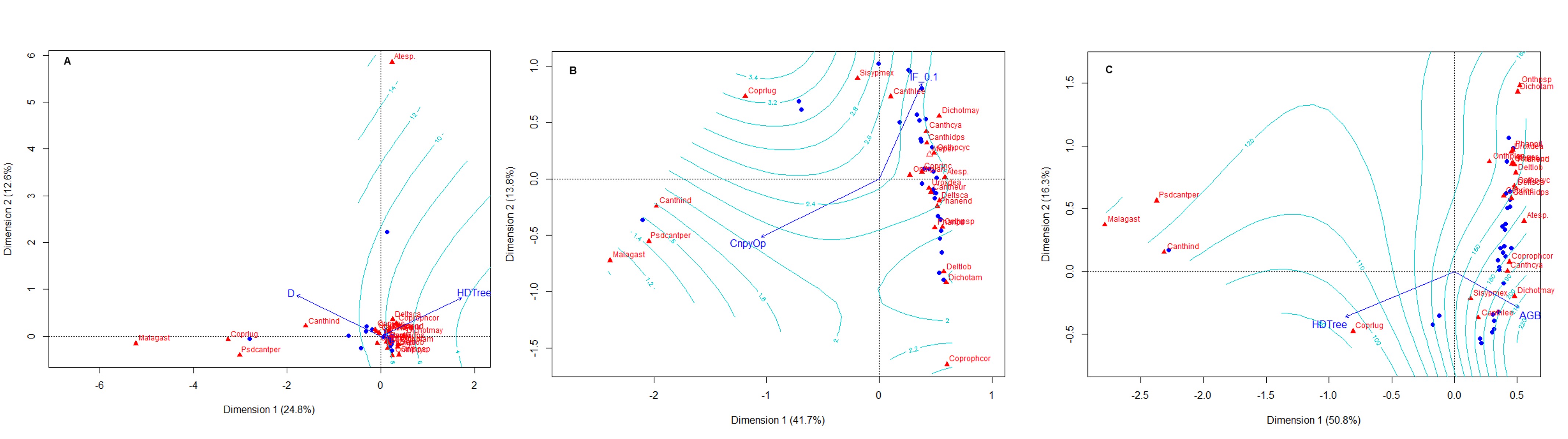

Supplement: S3 Fig — The vectors represent the direction of the most important variables of the forest in the chronosequence. D: Simpson’s diversity index; HDTree: Dominance tree height; CnpyOp: Canopy openness; IF0,1: Shannon exponential entropy-based inequality factor; AGB: Aboveground biomass. (JPG) [file pone.0337635.s007.jpg]
